# Development of high-performance two-dimensional gel electrophoresis for human hair shaft proteome

**DOI:** 10.1371/journal.pone.0213947

**Published:** 2019-03-19

**Authors:** Sing Ying Wong, Onn Haji Hashim, Nobuhiro Hayashi

**Affiliations:** 1 Department of Life Science and Technology, Graduate School of Life Science and Technology, Tokyo Institute of Technology, Meguro-ku, Tokyo-to, Japan; 2 Department of Molecular Medicine, Faculty of Medicine, University of Malaya, Kuala Lumpur, Malaysia; 3 University of Malaya Centre for Proteomics Research, University of Malaya, Kuala Lumpur, Malaysia; Shantou University Medical College, CHINA

## Abstract

The primary components of human hair shaft—keratin and keratin-associated proteins (KAPs), together with their cross-linked networks—are the underlying reason for its rigid structure. It is therefore requisite to overcome the obstacle of hair insolubility and establish a reliable protocol for the proteome analysis of this accessible specimen. The present study employed an alkaline-based method for the efficient isolation of hair proteins and subsequently examined them using gel-based proteomics. The introduction of two proteomic protocols, namely the conventional and modified protocol, have resulted in the detection of more than 400 protein spots on the two-dimensional gel electrophoresis (2DE). When compared, the modified protocol is deemed to improve overall reproducibility, whilst offering a quick overview of the total protein distribution of hair. The development of this high-performance protocol is hoped to provide a new approach for hair analysis, which could possibly lead to the discovery of biomarkers for hair in health and diseases in the future.

## Introduction

Proteomics has been extensively used over the last decade due to its ability to unveil the proteome of a cell, tissue, or organism and explicitly exhibit their dynamic states [1]. However, this high-throughput technique was seldom used to describe the proteome of human hair shaft.

The study of such simple and stable specimen has not been broadly discussed from the proteomics standpoint until recent years. This was due to the difficulty in extraction of hair protein caused by its high stability and keratin content, which often remains insolubilized even in strong denaturants [2]. These keratins, together with small hydrophobic proteins, known as keratin-associated proteins (KAPs), make up the primary structural of the human hair [3]. Besides, the insolubility of hair was also a result of the extensive disulfide bond cross-linking between KAPs and keratin intermediate filaments (KIFs), which exist in cytoplasm of hair cortical cells [4]. The interaction between KIFs and KAPs is not only crucial for hair growth, but also to provide tensile strength and elasticity to the hair [5, 6]

Several efforts have been made to extract hair protein, followed by identification of proteins present in human hair. To date, over 300 proteins were identified [7]. Nonetheless, the study of human hair shaft proteins by gel-based proteomics was scarcely reported. This may be due to several reasons, such as extremely biased composition to keratins as the major protein, inadequate amount of extracted hair protein, ineffectual solubilization of protein and/or incompatibility of processed sample with downstream applications such as isoelectric focusing (IEF) and two-dimensional gel electrophoresis (2DE).

In 2002, when “Shindai method” was reported as a convenient extraction methodology for human hair [8], interest in studying hair proteome aroused. The study also examined the composition of the extracted protein using 2DE and resulted in at least two spots in the more acidic region together with at least three spots from the acidic to basic regions [8]. Some proteins also appeared as unfocused horizontal streaks, which may be caused by the lack of sample clean-up and protein precipitation that contributed to high salt ions in the sample [9]. Hence, it is considered obligatory to remove potential impurities and interfering substances prior to sample application in order to attain desirable 2DE gel [10]. Even so, there is possible loss of protein during precipitation [11] that could eventually result in inadequate sample at undetectable level. Moreover, it is also believed that high-abundant proteins may impede the appearance of low-abundant proteins in the sample [12].

The study of post-translational modifications and protein abundances of hair is essential for the thorough understanding of human hair. This is feasible through the use of 2DE [13]. This classical method could separate proteins based on their charge as well as molecular size in order to generate protein expression profiles [14]. A representative view of hair complexity could be obtained, which would then be useful for comparative as well as protein expression analysis.

Overcoming the challenges in hair protein extraction is therefore imperative. The current study has selected an alkaline-based method to obtain hair proteins from hair shaft samples. Previous study has demonstrated efficient extraction of hair protein by heating short strands of hair in an alkaline lysis buffer [15]. Subsequently, the quality of the extracted protein by this methodology and its compatibility with the downstream processes were assessed in this study. Through the use of this method of extraction, adequate protein was attainable to be resolved in both first and second dimensional gel electrophoresis.

Following the separation of hair protein across a two-dimensional gel using conventional proteomic techniques, the present study then applied some modifications on these sophisticated techniques to overcome some limitations that were found in the conventional protocol. The comparison between these protocols including the difference in sample preparation, isoelectric focusing (IEF) as well as 2DE was made to determine the most suitable method to reveal information of such complex tissue—hair. The establishment of this high-performance human hair shaft proteomics is essential for comparative analysis in the future, where gel-to-gel reproducibility is often a point in question. In addition to providing an insight into hair proteome profile, the adoption of these high-throughput techniques in the modified protocol could serve as a novel approach to discover potential biomarkers of hair health, ageing and various diseases in the future.

## Materials and methods

Conventional proteomic techniques and protocol mentioned throughout this study were carried out in the Department of Molecular Medicine of University of Malaya, Malaysia [15].

### Hair protein extraction

Samples of hair shaft were collected from two unrelated healthy volunteer subjects (n = 2) without prior hair treatment such as hair dyes, perm and bleach. The hair samples were sterilized with 90% ethanol for lipid removal and extraction of hair protein was undertaken according to the protocol reported in the previous study [15], which involved the adoption of alkaline lysis buffer. However, the second stage of extraction was omitted in this study to reduce the total extraction duration. This study and its consent procedure were approved by the Ethical Committee of Tokyo Institute of Technology (Ref. no.: 2018071).

### Protein precipitation, solubilization and quantification

Samples were purified and concentrated using two different methods: acetone precipitation by mixing one volume of sample with four volumes of pre-cold acetone in the conventional protocol; or the use of 2D clean-up kit (GE Healthcare, FairField, CT) according to the manufacturer’s guideline in the modified protocol. An additional step was added to the default 2D clean-up protocol by dispersing the protein pellet in iced water using Ultrasonic Disintegrator (MU-8, Progen, London, UK) prior to incubating the tube at -20°C for at least 30 min. The precipitated protein was later reconstituted in either sample buffer [7 M urea; 2 M thiourea; 4% CHAPS; 2% ampholytes (IPG 4–7); 1% dithiothreitol (DTT)] or Destreak Rehydration Solution (GE Healthcare, Amersham, UK). Prior to applying samples for gel electrophoresis, the concentration of protein in both samples were determined by Bradford colorimetric method or 2D Quant kit (GE Healthcare, FairField, CT).

### Sample preparation for 2DE

Each hair protein solution of 100 μg was prepared for 2DE. In the conventional protocol, protein solution was mixed with rehydration solution which has similar constituent to the sample buffer prepared earlier, except a few grains of DTT was only added prior to use. A few grains of Orange G as tracking dye was also added. On the the other hand, hair protein solution was mixed with Destreak Rehydration solution (GE Healthcare, Amersham, UK) and 0.7 μL of IPG buffer (pH 3–10) in the modified protocol. Any undissolved substances were removed using a Spin Filter (Agilent Technologies, CA, USA). In both experiments, the volumes of protein solution added were based on the result of protein quantification. Moreover, different types of Immobiline DryStrips (13 cm, pH 4–7; 7 cm, pH 3–10) were also compared in this study. The former was adopted in the conventional protocol. In both experiments, the Immobiline DryStrips were incubated in the sample mixture on the Immobiline Drystrip Reswelling Tray (GE Healthcare, Uppsala, Sweden). Appropriate amount of Immobiline DryStrip Cover Fluid was added into the samples before the DryStrips were allowed to incubate for 18 h at room temperature (RT).

### Isoelectric focusing (IEF) of proteins

The conventional protocol performed IEF on Multiphor II Flatbed electrophoresis system and Electrophoresis Power Supply EPS-3501 XL (GE Healthcare, Uppsala, Sweden). It was performed by gradually increasing the voltage across the DryStrip under the following conditions: (i) 0–500 V, 1 h; (ii) 1000 V, 1 h; (iii) 8000 V, 2 h 30 m, and (iv) 8000 V, 55 m. When the run was complete, the focused strip was stored at -80°C in screw-cap tubes for overnight. For the modified protocol, first-dimensional separation of protein was done using similar power supply, except Multiphor II (GE Healthcare) and a cooling circulator (Julabo, Seelbach, Germany) was linked. The conditions used to run IEF also differed: (i) 0–300 V, 1 min; (ii) 300 V, 1.5 h; (iii) 300–3500 V, 1.5 h and (iv) 3500 V, 500 hours (= ∞). It was allowed to run for at least 5 hours until the current value became constant. The strip was immediately used for the next step.

### Equilibration of immobiline DryStrips

The DryStrip kept at -80°C was first equilibrated in equilibration buffer [6 M urea; 0.05 M Tris-Hydrochloric Acid (Tris-HCl), pH 8.8; 30% v/v glycerol; 2% sodium dodecyl sulfate (SDS); a few grains of bromophenol blue] with 1% w/v DTT, then in equilibration buffer with 4.5% w/v Iodoacetamide (IAA) for 20 m each at RT. The modified method also utilized equilibration buffer with the same components, except the concentrations of Tris-HCl and glycerol were altered to 1.5 M and 30% v/v, respectively. Moreover, the DryStrip was only incubated for 15 m each at RT.

### 2DE of proteins

The comparison between hand-cast and pre-cast gels, as well as different SDS-PAGE gel percentages and sizes were also made in this study. The conventional protocol involved hand-casting gradient gel of 8–15%. Glass plates of 16 x 18 cm, with a 1 mm thick gradient gel was prepared using a gradient maker (Model SG 30, Hoefer, USA). Agarose sealing solution of 0.5% was used and electrophoresis was performed by SE 600 Ruby Electrophoresis System (GE Healthcare, Uppsala, Sweden) linked to a cooling circulator (Grant Instrument Ltd., Cambridge, UK) and Power Supply-EPS601 (GE Healthcare) at 18°C. The gel was run using SDS electrophoresis buffer (25 mM Tris; 198 mM glycine; 0.1% SDS) under these conditions: (i) Phase 1: 50 V, 150 mA, 100 W for one hour; (ii) Phase 2: 600 V, 150 mA, 100 W until the tracker dye reached the bottom of the gel. In the modified protocol, pre-cast NuPAGE 4–12% Bis-Tris ZOOM Gel, size of 8 x 8 cm (Invitrogen, California, USA) was used and electrophoresis was performed in the Mini-PROTEAN Tetra Vertical Electrophoresis Cell (Bio-Rad). Similarly, 0.5% agarose sealing solution was used, but NuPAGE MOPS SDS Running Buffer (1X) was used to run the electrophoresis at 200 V, 2 mA instead. The electrophoresis was stopped when the marker reached the mark above the protrusion of the gel cassette.

### Gel-staining

Two different gel-staining methods were compared in the current study: silver-staining for conventional protocol; Sypro Ruby-staining for modified protocol. As for silver-staining protocol, gel was first fixed with fixation solution (40% v/v ethanol; 10% acetic acid) for 30 m, then incubated in sensitizing solution (30% v/v ethanol; 0.5 M sodium acetate trihydrate; 12.7 mM sodium thiosulphate) for another 30 m before washing with distilled water. The washing step was repeated thrice for 5 min each. Next, the gel was impregnated with silver solution (14.7 mM silver nitrate) for 20 m, then rinsed twice with Milli-Q water for 1–2 m to remove excess silver solution. The gel was later incubated in developing buffer (0.24 M sodium carbonate; 0.04 v/v formaldehyde) for image development. When sufficient degree of spot intensities has been achieved, staining is stopped using stop solution [40 mM ethylenediaminetetraaacetic acid (EDTA) sodium dihydrate] for 30 min before rinsing twice with distilled water. This silver-staining method was performed on an orbital shaker (BioLab, UbiTechPark, Singapore) which was set at a constant speed of 50 rpm. The gel was then finally scanned using ImageScanner III. In comparison with the conventional protocol, Sypro Ruby Protein Gel Stain (Lonza Rockland, ME, USA) was selected for the modified protocol in this study. Gel obtained from 2DE was fixed with fixation solution (50% methanol; 7% acetic acid) for 30 m for twice, then stained with 40 mL of Sypro Ruby Gel Stain in a shielded state for overnight. The gel was later washed with Milli-Q water and destained with decoloration solution (10% methanol; 7% acetic acid). Finally, the solution was replaced with Milli-Q water and gel was scanned using Typhoon FLA 9000 Scanner (GE Healthcare, Uppsala, Sweden). Gels obtained from both protocols were analyzed using ImageMaster 2D Platinum Software.

## Results

### Quantification of hair protein

It is crucial to evaluate the appropriate assay of protein quantification, as well as the method for sample clean-up to suit the experimental design in this study. The conventional protocol utilized pre-cold acetone for protein precipitation and resulted in 5.68 μg/μl when quantified using Bradford colorimetric assay. On the other hand, in the modified protocol, 2D Clean-up kit was used and 2D Quant kit has quantified 6 μg/μl of extracted protein. Although the amount of extracted proteins in both experiments were comparable, the efficacy of each clean-up method should be conferred altogether with the resulted 2DE gel profiles, which will be further discussed later in this study.

### Resolubilization of hair protein pellets

In both experiments prior to quantification, protein pellets were reconstituted in either sample buffer or Destreak Rehydration solution. When sample buffer containing DTT was used, the protein pellets were completely dissolved. On the other hand, in the case where Destreak Rehydration solution was used, some parts of the protein pellets obtained from 2D cleaned-up sample remained jelly-like, which was resistant to solubilization.

### Hair protein expression profiles

**Fig 1:**
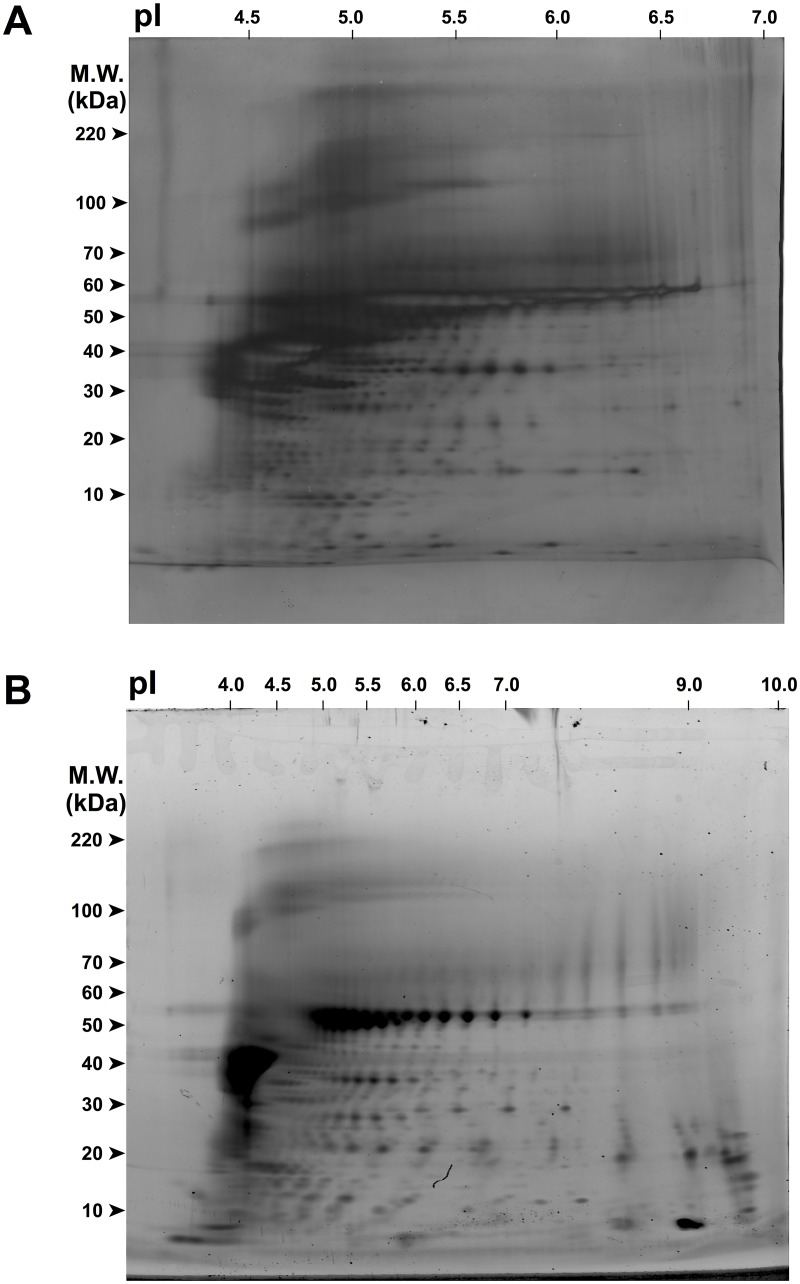
2DE gel protein expression profiles of human hair shaft protein. (A) Conventional protocol. Proteins were separated in the first dimension on an IPG strip (pH 4–7; 13 cm) and in the second dimension on a hand-cast 8–15% gradient gel. Gel was silver-stained and scanned using Image Scanner III. (B) Modified protocol. Proteins were separated in the first dimension on an IPG strip (pH 3–10; 7 cm) and in the second dimension on a pre-cast 4–12% gradient gel. Gel was Sypro Ruby-stained and scanned using Typhoon FLA 9000 Scanner. Both gels were loaded with 100 μg of hair protein with reference to result obtained from protein quantification.

The current study has successfully obtained 2DE gel profiles of human hair through both the conventional and modified protocols. The distribution of spots of hair proteins is displayed in Fig 1. Spot detection was also performed on these gels using Image Master 2D Platinum Software and the result is displayed in Table 1. In the modified protocol, the number of protein spots was increased by approximately 10% when compared to the conventional protocol.

**Table 1:** Number of protein spots detected in both conventional and modified protocol.

Protocols	No. of spots	Staining
Conventional	452	Silver
Modified	496	Sypro Ruby

In the conventional protocol, silver-staining method was used; in the modified protocol, Sypro Ruby staining was used. Both scanned gels were analyzed using Image Master 2D Platinum Software.

## Discussion

Although several reports have identified proteins in hair using other technologies, these reports lacked the vigor of a 2DE analysis that include exemplary quantitation in combination with separation of protein variants [16]. Therefore, 2DE is still a favored technology for the analysis of human hair shaft in many means.

Hair protein extracted using alkaline-based method was shown to be compatible with the downstream application used in this study. This explains that the use of high temperature together with alkaline lysis buffer were able to break the highly cross-linked protein networks in hair [15], causing this complex mixture to be well separated in the first and second dimensional gel electrophoresis. Since the intensities of spots is proportional to the quantity of the protein on the gel [17], the high-abundant proteins based on the expression profiles were observed mostly in the acidic region with molecular weight of 30–60 kDa. These proteins were thought to be largely acidic type I keratins because of their pI value which ranges from 4.5–5.5 [18].

When spot number was taken into account, there was an increase in spot detection in the modified protocol, which could be due to sample variability and/or improvements in the proteomics techniques used. In both experiments however, the number of protein spots detected was greatly increased when compared to previous study using “Shindai method”, where only at least 5 protein spots were reported in the acidic and neutral to basic regions [8]. It is evident that the use of alkaline-based method together with a high-performance sample processing protocol is essential to obtain desirable 2DE gel profile of hair protein. Furthermore, the hair protein expression profile obtained in the current study shows similar pattern to the 2DE gel profile of wool [19], where the high abundant proteins were identified as type I keratins in the acidic region and type II keratins in the neutral to basic region. Although it was not known the total number of protein spots, it was noted that low molecular weight proteins of wool being resolved in the 2DE gel were sparse. The detection of these low molecular weight human hair proteins in this study could reveal the small hydrophobic proteins in hair, known as keratin-associated proteins with a typical molecular weight of 6–30 kDa [20]. In addition, it is also believed that some non-keratin proteins present in hair were also isolated altogether in this study. Nonetheless, since keratins and keratin-associated proteins are the primary components of human hair, these proteins tend to dominate 2DE map, hindering the detection of less abundant proteins [12]. Future work should be done in order to reveal these non-keratin proteins in hair.

Considering the number of protein spots detected in both conventional and modified protocol did not vary greatly, the overall performances of the protocols were further compared in order to determine the most suitable protocol for hair proteome analysis. The comparison was made based on several factors such as gel resolution, reproducibility, total time and effort required. Much work has been reported emphasizing on the efficacies of different clean-up methods. The 2D Clean-up kit used in the modified protocol utilized TCA/acetone precipitation of proteins for the removal of the sample proteins from interfering substances such as salts, lipids, nucleic acids, etc [21]. Similarly, acetone precipitation has also been proven to be an inexpensive and easy alternative which also results in high protein yield and presumably more spots in 2DE gel [22]. However, the present study showed that the use of 2D Clean-up kit in the modified protocol has resulted in slightly more spots than in the conventional protocol (Table 1). With that said, it should also be taken into account that this could be due to difference in samples used. Additionally, it was also noteworthy that the use of acetone precipitation has collected majority of the hair proteins and failed to remove some of which are high in abundance, mostly in the acidic region, with pI value close to 4.5. It is therefore implied that 2D Clean-up kit is a better option for hair protein concentration, which was also able to leave out part of the high-abundant hair proteins and generated clearer display of proteins together with their isoforms on the gel profile.

After isolation of protein from hair shaft, protein pellets were reconstituted in either sample buffer or Destreak Rehydration solution, where both buffers contained denaturing agents such as urea and thiourea. Since the concentration of these two reagents used in both experiments were identical, the incomplete solubilization of protein pellets in Destreak Rehydration solution suggests that the addition of thiol reducing agent, DTT, used in the sample buffer of conventional protocol has successfully disrupted the intramolecular and intermolecular disulfide bonds of hair protein [23]. This allowed the hair protein to unfold and retain in its fully reduced state. However, the use of DTT during IEF has been reported to cause unsatisfactory 2DE gel profile, such as unfocused and disappearance of some spots [24]. Therefore, the suitability of each sample buffer is also further assessed in adjacent to the generated 2DE gel profile of hair protein. Despite the incomplete solubilization of hair protein pellet in Destreak Rehydration solution used in the modified protocol, more protein spots were detected (Table 1). Moreover, streaking was also remarkably improved in the generated 2DE gel profile (Fig 1). This was due to its ability to prevent unspecific oxidation of protein thiol groups [25, 26]. Other than improved focusing of proteins, spot pattern of 2DE gel profile of hair sample solubilized in Destreak Rehydration solution is regarded to be simpler.

There are various types of IPG immobiline drystrips available in the market. Thus, the evaluation of a suitable IPG immobiline drystrip for separation of hair proteins was also performed in this study. Generally, choice of length and pH range of strips should be dependent on the protein of interest of a study [27]. The use of narrower pH range, such as the one used in the conventional protocol (pH 4–7), should result in better separated protein species and isoforms due to the expansion of a small pH range across the entire width of a gel [28]. Even so, the use of wide pH range (pH 3–10) in the modified protocol has resulted in more protein spots detection, whilst providing a broad overview of total protein distribution in human hair. Besides, it was able to resolve more hair proteins in the neutral to basic region, which may have contributed to the total number of protein spots. These proteins were thought to be type II basic keratin due to their pI value of 6.5–7.5 [18]. Hence, the use of IPG strip with pH 3–10 is much preferred in the current study, in alignment with its aim to serve as a preliminary study on proteins of human hair shaft through gel-based proteomics. A quick overview of the total hair protein is attainable when IPG strip shorter in length and broader in pH range is used.

The conventional and modified protocol in the current study have selected gradient gel for its advantages to estimate the complexity of hair protein and analyze wide range of protein sizes [29]. After interesting region of 2DE has been determined, single acrylamide percentage gel may be run in the future to study hair proteins of particular size range. For high-throughput application, pre-cast gel is often preferred due to its ability to save time, labor and improve gel-to-gel reproducibility for their comparisons [30]. In the current study, it was noted that quality control remains difficult in hand-cast gel used in the conventional protocol to some extent. Moreover, smaller gel size used in the modified protocol as opposed to the conventional protocol, clearly has reduced the total running and analysis time by at least half, without compromising the gel quality.

Following electrophoretic separation of hair proteins, the choice of image analysis method is examined, specifically between silver-staining and Sypro Ruby staining. Very often, the staining method is closely reliant to the intended downstream analytical procedures. Other than being a more sensitive staining method than silver-staining, Sypro Ruby has also proven to demonstrate improved linearity, batch-to-batch consistency and enhanced recovery of peptides from in-gel digests for MALDI-TOF mass spectrometry [31]. Besides, silver-stained gel has resulted in some “negative spots” and/or poorly-stained spots of high-abundant hair proteins of 35–55 kDa in the acidic region (Fig 1a). These spots often appear during early stage of development and are strongly dependent on the silver concentration used [32]. This could be solved by longer development times but it would also risk over-staining of the gel, especially in the region of high-abundant proteins. Hence, when consistency is a critical parameter, Sypro Ruby staining used in the modified protocol is favored for proper visualization of hair proteins in this study.

Other than the reasons such as use of 2D Clean-up kit and pre-cast polyacrylamide gel, another key parameter that contributed to the overall increased reproducibility is the downsized polyacrylamide gel employed in the modified protocol. When proteins are separated in the two dimensions in such short separation distance, the electrical field strength applied is maximized. Not only it allows separations of proteins in a short period of time, the electric field passing across the downsized gel could easily be kept consistent. Moreover, temperature control, which is another essential parameter to yielding high resolution gel, could also be achieved when using gel in smaller size.

Present findings offer the prospect of several valuable directions for future hair shaft protein analysis. Both the conventional and modified protocol used in this study were able to resolve hair proteins well in the electrophoretic separation, with the aid of an effective extraction method, known as the alkaline-based method. In the later part of this study, the comparison between these protocols was able to help determine the most suitable techniques to be used for future hair protein analysis. An overview of the different experimental procedures used in both of the protocols and the basis of each modification is also shown in Table 2. Overall, the modified protocol was proven to be less laborious, sensitive, rapid and easily applied in the current study. The development of this high-performance human hair shaft proteomics is anticipated to serve as a fundamental guideline for future scientists to conduct various studies on human hair shaft, potentially including biomarkers studies related to hair health, diseases and ageing.

**Table 2:** Overview of the different experimental procedures used in both conventional and modified protocol.

Experimental procedures	Protocols		Basis of modifications
	Conventional	Modified	
Removal of impurities from samples	Acetone precipitation	2-D Clean-Up kit	The 2-D Clean-Up kit was effective in removing impurities from samples by utilizing both precipitant and co-precipitant to quantitatively precipitate proteins. The precipitated protein pellet was later washed again with wash buffer, which could further remove nonprotein contaminants.
Resolubilization of proteins	Sample buffer	Destreak Rehydration solution	The presence of DTT in the sample buffer could completely resolubilized acetone-precipitated protein but could cause unfocused spots. Hence, in the modified protocol, Destreak rehydration solution was used. It could reduce streaking while producing focused spots due to its ability to prevent unspecific oxidation of protein thiol groups.
First dimension protein separation	IPG immobiline dry strip pH 4–7, 13 cm	IPG immobiline dry strip pH 3–10, 7 cm	Shorter strip with wider pH range was used in the modified protocol to obtain a quick overview of the total hair protein distribution of the sample.
Second dimension protein separation	8–15% Hand-cast gradient gel; 16 x 16 cm	4–12% Pre-cast gradient gel; 8 x 8 cm	The use of pre-cast gel is not only convenient, but quality control is achievable. The downsized gel in the modified protocol is also able to ensure consistent temperature and electrical field passing across the gradient gel when running 2DE.
Gel-staining method	Silver	Sypro Ruby	Sypro Ruby shows less protein-to-protein variability, when compared to silver-stain. When proteins are silver-stained, some protein spots could not be stained at all, or are seen as “hollow spots” when visualized. Sypro Ruby, on the other hand, binds to basic amino acids as well as the polypeptide backbone. Moreover, it has extremely high staining capacity where it stains most classes of proteins including those which are challenging to stain.

This includes the different methods used when removing impurities from hair samples, re-solubilization of proteins, separation of proteins and staining of the generated 2DE gels. The basis of modifications was also included to demonstrate the factors of the increased gel resolution and improved reproducibility in the modified protocol, which were difficult to achieve in the conventional protocol.
